# Tomotherapy as an adjuvant treatment for gastroesophageal junction and stomach cancer may reduce bowel and bone marrow toxicity compared to intensity-modulated radiotherapy and volumetric-modulated arc therapy

**DOI:** 10.18632/oncotarget.14459

**Published:** 2017-01-03

**Authors:** Xin Wang, Yuan Tian, Yuan Tang, Zhi-Hui Hu, Jia-Jia Zhang, Gui-Shan Fu, Pan Ma, Hua Ren, Tao Zhang, Ning Li, Wen-Yang Liu, Hui Fang, Ye-Xiong Li, Jing Jin

**Affiliations:** ^1^ Department of Radiation Oncology, Cancer Hospital and Institute, National Cancer Center, Chinese Academy of Medical Sciences and Peking Union Medical College, Panjiayuan Nanli, Beijing, P. R. China

**Keywords:** gastroesophageal junction cancer, gastric cancer, helical tomotherapy, intensity-modulated radiotherapy, volumetric-modulated arc therapy

## Abstract

**Purpose:**

To compare dosimetric parameters of intensity-modulated radiotherapy (IMRT), volumetric-modulated arc therapy (VMAT) and tomotherapy (TOMO) in the adjuvant treatment of gastroesophageal junction (GEJ)/stomach cancer. The planning goal was to maintain high target coverage while keeping the dose to the bowel and bone marrow (BM) as low as possible.

**Materials and Methods:**

After curative surgery, 16 patients with GEJ/stomach cancer were re-planned by coplanar IMRT (five fixed beam), VMAT (double-arc), and TOMO. The dose to the planning target volume (PTV) was 45 Gy in 25 fractions. The target parameters, including the homogeneity index (HI) and conformity index (CI), and doses to the organs at risk (OARs) were analyzed.

**Results:**

Dosimetric parameters for PTV and OARs were comparable among the three techniques. However, TOMO provided improved conformity (CI = 0.92±0.03) and homogeneity (HI = 1.07±0.02) than IMRT (CI = 0.87±0.03; HI = 1.09±0.02; *p* < 0.05) and VMAT (CI = 0.86±0.03; HI = 1.09±0.02; *p* < 0.01). TOMO also improved dose sparing of the bowel (percentage of the volume receiving a dose of ≥ 30 Gy [V30] = 23.24±9.85) and BM (V30 = 71.66±6.15) compared with IMRT (bowel V30 = 30.02±11.74; BM V30 = 83.74±8.42; *p* < 0.01) and VMAT (bowel V30 = 31.88±11.59; BM V30 = 79.51±9.07; *p* < 0.01).

**Conclusions:**

TOMO is a good option for adjuvant treatment of GEJ/stomach cancer in patients undergoing radical surgery due to its superior bowel and BM dose sparing, dose conformity and dose homogeneity; however, future studies are required to validate its clinical efficacy.

## INTRODUCTION

The intergroup 0116 (INT-0116) study in 2001 established the role of fluorouracil-based adjuvant chemoradiotherapy (ACRT) in the treatment of high risk, completely resected adenocarcinomas of the gastroesophageal junction (GEJ) and the stomach [1, 2]. However, a high incidence of acute gastrointestinal (GI) and hematologic adverse events often results in delayed or missed chemotherapy or radiotherapy treatments, which may impact patient prognosis. Thus, it is important to reduce the probability of GI and hematologic problems by reducing the irradiation dose to the bowel and bone marrow (BM). With the more advanced radiation techniques now available, it has become possible to deliver such reduced doses.

Several studies have proved that intensity-modulated radiotherapy (IMRT) is superior to two-dimensional radiotherapy (2D-RT) or 3D-RT, as it provides a more conformal and homogeneous dose to the planning target volume (PTV), thus minimizing the probability of toxicity [3–5]. In recent years, implementation of volumetric-modulated arc therapy (VMAT) and tomotherapy (TOMO) is gaining traction, as their dosimetric performance rivals that of IMRT [6–9]. However, to date, no study has compared the IMRT, VMAT and TOMO plans in the adjuvant treatment for adenocarcinomas of the GEJ and the stomach.

Our objective was to compare IMRT, VMAT and TOMO with regards to the PTV and organs at risk (OARs) dosimetric parameters for adjuvant treatment of GEJ and stomach cancer. The planning goal was to maintain high target coverage while keeping the dose to the bowel and BM as low as possible.

## RESULTS

### IMRT, VMAT, and TOMO comparison based on the planning target volume

**Figure 1:**
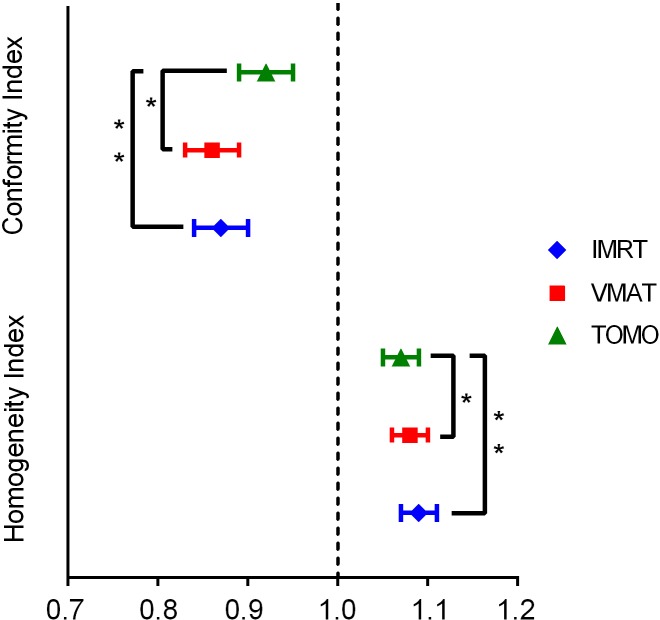
Conformity index (CI) and homogeneity index (HI) for planning target volume (PTV) with IMRT (rhombus), VMAT (square), and TOMO (triangle)

**Figure 2:**
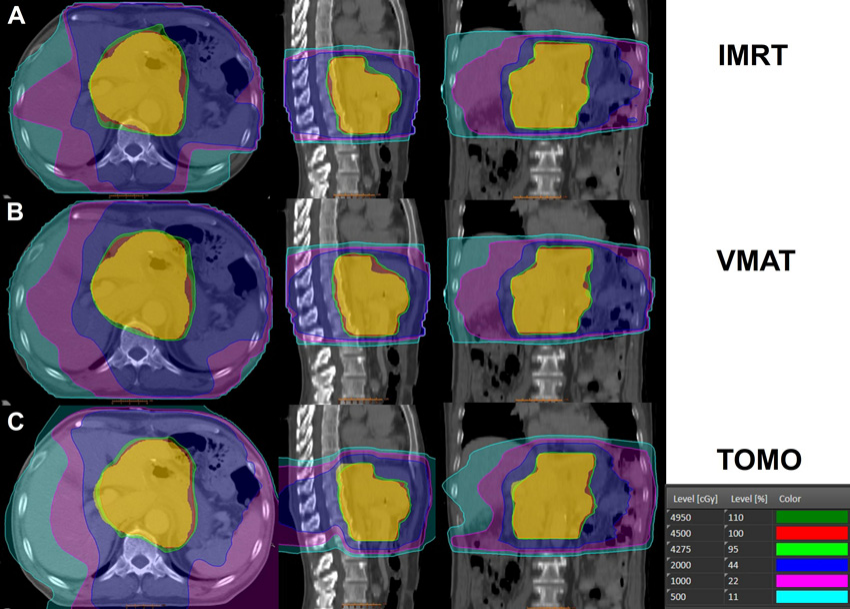
**Examples of planning target volume (PTV) dose distributions used for a. IMRT, b. VMAT, and c. TOMO in a patient who underwent distal partial gastrectomy**.

The mean volume of the PTV was 960.61 ± 287.59 cm^3^. Detailed results of dosimetric comparison for the PTV are shown in Table 1. Among the three plans, TOMO showed the best dose conformity and homogeneity compared to IMRT (*p* < 0.05) and VMAT (*p* < 0.01, Figure 1). The mean dose (D_mean_) to the PTV was acceptable for all treatment plans, with no statistically significant differences between the three techniques. Figure 2 shows examples of PTV dose distributions obtained for IMRT, VMAT and TOMO plans in a patient who underwent distal partial gastrectomy.

**Table 1:** Dosimetric comparison for PTV and OARs and quick reference value for advantages among three techniques (based on *P* value)

Parameter		Mean ± SD			Quick reference guide (*P* value)		
		IMRT	VMAT	TOMO	IMRT *vs* VMAT	IMRT *vs* TOMO	VMAT *vs* TOMO
PTV	D_mean_ (Gy)	46.43±0.25	46.63±0.26	46.36±0.30	IMRT*	—	TOMO*
	PTV_95_ (%)	99.30±0.41	99.36±0.28	99.47±0.37	—	—	—
	PTV_100_ (%)	95.10±0.35	95.00±0.63	95.51±1.29	—	—	—
	PTV_105_ (%)	12.85±12.27	27.87±17.98	9.66±11.01	IMRT^*^	—	TOMO^*^
	PTV_110_ (%)	0.02±0.06	0.19±0.58	0	—	—	—
	HI	1.09±0.02	1.09±0.02	1.07±0.02	—	TOMO*	TOMO^*^
	CI	0.87±0.03	0.86±0.03	0.92±0.03	—	TOMO*	TOMO^*^
Bowel	V_20_ (%)	56.82±14.66	58.04±12.98	51.49±14.27	—	—	TOMO*
	V_30_ (%)	30.02±11.74	31.88±11.59	23.24±9.85	—	TOMO^*^	TOMO^*^
	V_40_ (%)	11.91±5.86	12.09±5.94	9.58±4.72	—	TOMO^*^	TOMO^*^
	V_45_ (%)	6.28±3.66	6.15±4.63	5.11±3.38	—	TOMO^*^	TOMO^*^
	D_1_ (Gy)	47.47±1.70	47.14±1.64	46.36±1.45	—	TOMO^*^	TOMO^*^
BM	V_5_ (%)	97.43±4.04	98.06±3.70	98.39±2.35	IMRT*	—	—
	V_10_ (%)	95.03±5.56	95.08±5.33	94.11±3.89	—	—	—
	V_20_ (%)	93.21±5.85	91.41±5.70	84.73±4.36	VMAT^*^	TOMO^*^	TOMO^*^
	V_30_ (%)	83.74±8.42	79.51±9.07	71.66±6.15	VMAT^*^	TOMO^*^	TOMO^*^
L-Kidney	V_5_ (%)	78.00±7.48	87.66±7.16	89.95±7.78	IMRT^*^	IMRT^*^	—
	V_20_ (%)	22.67±1.86	19.11±2.08	22.89±5.87	VMAT^*^	—	VMAT*
	D_mean_ (Gy)	13.98±1.38	14.47±1.04	14.63±2.64	—	—	—
R-Kidney	V_5_ (%)	85.68±10.68	81.58±10.88	79.16±14.51	VMAT^*^	TOMO*	—
	V_20_ (%)	17.45±4.27	18.11±3.55	18.91±5.24	—	—	—
	D_mean_ (Gy)	13.60±1.57	13.78±1.59	12.91±2.31	—	—	—
Liver	V_5_ (%)	98.18±1.89	98.68±1.22	91.66±4.80	IMRT*	TOMO^*^	TOMO^*^
	V_30_ (%)	22.02±3.93	21.41±4.56	22.29±5.58	—	—	—
	V_40_ (%)	13.33±4.48	13.40±4.60	12.60±5.11	—	TOMO*	TOMO*
	D_mean_ (Gy)	19.49±1.69	20.22±1.82	17.97±2.21	IMRT^*^	TOMO^*^	TOMO^*^
PRV SC	D_1_ (Gy)	33.69±3.95	33.95±2.42	34.61±2.17	—	—	—
MUs		524±102	419±48	5381±966	VMAT^*^	IMRT^*^	VMAT^*^

Abbreviations: BM, bone marrow; CI, conformity index; D_1_, the minimum dose that 1% of the volume of the organ receives; D_mean_, mean dose; HI, homogeneity index; IMRT, intensity-modulated radiotherapy; MUs, monitor units; PRV SC, planning organ at risk volume of the spinal cord; PTVn: percentage of the planning target volume receiving n% of prescription dose; SD, standard deviation; TOMO, tomotherapy; VMAT: volumetric-modulated arc therapy; Vn: percentage of the volume receiving ≥n Gy

*p <0.05; ^*^p <0.01

### IMRT, VMAT, and TOMO comparison based on the organs at risk parameters

**Figure 3:**
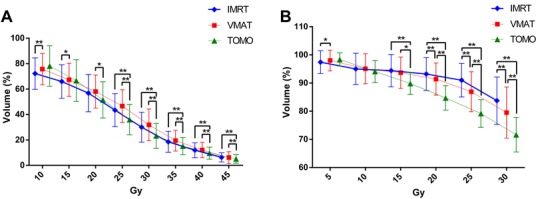
The mean dosimetric indices for IMRT (rhombus), VMAT (square), and TOMO (triangle) **a**. Illustration of the mean dosimetric indices for the bowel volume receiving 10-45 Gy with the three techniques. **b**. The bone marrow volume receiving 5-30 Gy with the three techniques.

The dose-volume histogram (DVH) data for the OARs are listed in Table 1. TOMO resulted in a lower dose to the bowel than IMRT and VMAT, which is best seen at the V30 (the V30 is the percentage of the volume receiving a dose of 30 Gy or more), V40, V45 and D1 (D1 is the minimum dose that 1% of the volume of the bowel receives) and corresponds to an approximately 20% reduction (*p* < 0.01, Figure 3a). Furthermore, TOMO significantly improved BM dose sparing for the V20 and V30, with a 8-15% reduction compared to the other two techniques (*p* < 0.01, Figure 3b). With regards to the liver, the V5, V40 and D_mean_ were significantly lower with TOMO (~5-11% reduction) compared to IMRT and VMAT (*p* < 0.05). In the left kidney, VMAT decreased the V20 (by ~16%) compared to TOMO and IMRT (*p* < 0.05). No significant advantage for one technique over the others was observed when examining the right kidney and the D1 of the planning organ at risk volume (PRV) of the spinal cord.

### Comparison of monitor units between IMRT, VMAT, and TOMO

The mean numbers of Monitor Units (MUs) required to deliver both IMRT and VMAT were significantly less than the number required for TOMO (*p* < 0.01, Table 1). The number of MUs required for VMAT was also significantly lower than those for IMRT (*p* < 0.05, Table 1).

## DISCUSSION

Our results indicated that TOMO resulted in the best dose conformity and homogeneity, as well as improvements of the bowel and BM sparing, in the adjuvant treatment of GEJ and stomach cancer.

In China, chemoradiotherapy is still considered as part of the adjuvant setting for upper GI cancer, as it is the traditional Chinese view that surgery should be the primary therapeutic option. However, the 2D-RT technique has been shown to contribute to severe hematologic and GI toxicities. Indeed, the reported incidences of Grade 3 or higher hematologic and GI toxicities are 54% and 33%, respectively [1]. The high incidences of acute GI and hematologic adverse events often result in delayed or missed chemotherapy treatments, which in turn, may impact on the patient prognosis. Therefore, reducing the irradiation dose to the bowel and BM is important for reducing the likelihood of these adverse events. Being one of the few hospitals in China that is simultaneously equipped with several advanced linear accelerators, we are in the unique position of being able to produce high quality radiotherapy plans of IMRT, VMAT and TOMO. Therefore, it is important for us to identify the relative merits and demerits of these individual techniques. Thus, we performed a detailed comparison of the IMRT, VMAT, and TOMO plans in patients with GEJ and stomach cancer in this study.

IMRT, VMAT, and TOMO are currently used for the treatment of cancers. Previous studies have examined the dosimetric differences relating to adjuvant radiation for gastric cancer by comparing IMRT/VMAT/TOMO with 3D-RT [4, 5, 10–15]. Compared with 3D-RT, IMRT/VMAT/TOMO techniques achieve comparable PTV coverage and may improve sparing of the OARs. For example, Minn et al. [5] found that IMRT might improve the dose sparing of the kidneys and the liver compared with 3D-RT. Moreover, Wang et al. [11] also reported that VMAT and IMRT plans achieved better dose distribution and improved the dose sparing of the left kidney and liver compared with 3D-RT.

When comparing IMRT with VMAT, Li et al. and Zhang et al. [7, 14] indicated that the VMAT technique might provide better protection for the kidneys than IMRT. Wieland et al. [8] reported that both TOMO and IMRT techniques could deliver efficient doses to the PTV, while delivering a lower dose to the kidneys. Similarly, Dahele et al. [6] reported that TOMO achieves better dose homogeneity for PTV coverage than IMRT, but that both TOMO and IMRT improve kidney or liver sparing. Our results were overall consistent with the above studies. We found that TOMO was associated with greater dose conformity and better dose homogeneity compared with IMRT and VMAT. This result might be explained by the fact that TOMO may provide better dose distribution for targets with longer and more complex shapes. Indeed, as the surgical resection for proximal partial gastrectomy or total gastrectomy in patients with GEJ or stomach lesions usually extends far into the chest, they require substantially longer and more complex irradiation fields compared to those patients with a distal partial gastrectomy. Therefore, TOMO may be more favorable in patients with proximal partial gastrectomy or total gastrectomy.

In the present study, we also found that TOMO could improve the V5, V40, and mean dose of the liver. Moreover, the VMAT technique showed the greatest reduction in the V20 of the left kidney. However, to date, no other studies have examined the dose distribution in these organs when examining ACRT of the bowel and BM.

Regarding the OARs, the bowel is one of the most important organs in such dosimetric studies because GI toxicity is a major concern to clinicians. Early studies indicated that a strong dose-volume relationship exists between the volume of irradiated bowel and acute toxicities for rectal cancer, prostate cancer, or endometrial cancer [16–18]. Exclusion of the remnant stomach from the radiation field may significantly reduce the severe GI toxicity without compromising long-term survival rates [26]. However, for adenocarcinomas of the GEJ or the stomach, there are currently no standard implications of the bowel contouring on CT based planning, let alone an optimal threshold dose volume of the bowel that can be irradiated, especially when the remnant stomach is excluded from the radiation field. In this study, we opted to contour the small intestine and the colon with the bowel loops together as the “bowel”. This is because the dose constraints for both the small intestine and the colon are similar, and it allows us to perform the procedure more easily and quickly. Using this technique, TOMO produced a significant dose reduction of approximately 20% of the V30, V40, and V45 over IMRT and VMAT. However, since there were limited cases in this study and numerous studies have shown conflicting parameters for predicting of GI toxicity [16–18], the clinical benefits of TOMO for reducing GI toxicities must be confirmed in further randomized or prospective studies.

With regards to hematologic toxicities, more than 50% of the activity of the BM is located in the thoracic and lumbar vertebra and pelvic bones [19], which is just within or near the treatment field in gastric cancer. Most studies have found that myelosuppression was significantly associated with the volume of the pelvic BM and lumbosacral BM receiving 10 and 20 Gy of radiation in pelvic cancer [20, 21]. Moreover, chemoradiotherapy had been recommended for locally advanced adenocarcinomas of the GEJ and the stomach [22–24], which may aggravate the damage to BM stem cells and result in severe hematologic toxicities compared to radiotherapy alone. Therefore, reducing the volume of BM receiving low-dose radiation might prevent hematologic toxicities. Indeed, several studies have identified that techniques designed to reduce BM irradiation, such as IMRT, in comparison with conventional radiotherapy, might reduce hematologic toxicities in gynecologic cancer during chemoradiotherapy [25, 26]. However, whether the application of VMAT or TOMO could reduce the BM dose compared to IMRT had not been investigated in patients with GEJ or stomach cancer. Our study shows that TOMO reduces the BM V20 and V30 by 8-15% compared to IMRT and VMAT in these patients. Furthermore, as the distribution of active and inactive BM is significantly different among individuals, it has been suggested that functional imaging techniques, such as magnetic resonance imaging or positron emission tomography, should be used to identify the active BM [27, 28]. However, due to economic reasons and low-level evidence of the studies above, we did not perform these functional imaging techniques in our study. Instead, we outline the whole vertebra as a reference for the BM.

There are some limitations of our study to be considered. First, a respiratory gating technique was not used as this is not the standard protocol in our hospital, and thus, its influence on the dose distribution was not investigated. Second, the planning time was not addressed due to the difficulty of recording the exact amount of time spent on individual plans when the physicists might be involved in different tasks at the same time. Finally, this is only a physical comparison of the three treatment plans. Therefore, further validation of the clinical relationship between the bowel and BM sparing generated by TOMO and the extent of the toxicity decrease is required.

In summary, to the best of our knowledge, this is the first study to compare the IMRT, VMAT and TOMO techniques with regards to the PTV and OARs parameters in ACRT for adenocarcinomas of the GEJ and the stomach. All three techniques could be used in such patients to achieve comparable PTV coverage and sparing of the OARs. TOMO provides superior dose conformity and homogeneity, dose sparing of the bowel and BM, and fulfills the other OARs constraints to the liver and kidneys distributions compared with step-and-shoot IMRT and VMAT. In addition, VMAT has high delivery efficiency, which will benefit patients who cannot tolerate a long treatment time. Since different technical limitations and clinical requirements apply to the different radiotherapy techniques, the choice of approach should be determined on an individual basis. Our future studies will focus on validating the clinical efficacy and physical parameters delivered by TOMO.

## MATERIALS AND METHODS

### Patient eligibility and simulation

From January 2014 to December 2014, 16 patients were selected from an ongoing prospective phase II study of S-1 based ACRT for locally advanced adenocarcinomas of the GEJ and the stomach. A total dose of 45 Gy (1.8 Gy/fraction, 5 days/week) was delivered. S-1 was administered every weekday at a dosage of 80 mg/m^2^/d, based on results of our previous phase I study [29]. Patient characteristics were presented in Table 2. According to the 7th edition of the American Joint Committee on Cancer staging system [30], five patients with GEJ cancer were at stage III, and two and nine patients with stomach cancer were at stage II and III, respectively.

**Table 2:** Patient characteristics

Characteristic	*N*	%
Median (range)	55 (36-73)	
Men	13	81.3
Location of primary tumor GEJ Stomach	5 11	31.3 68.7
Surgery type Proximal partial gastrectomy Distal partial gastrectomy Total gastrectomy	5 7 4	31.3 43.7 25.0
Stage (AJCC 7^th^) II (GEJ, Stomach) III (GEJ, Stomach)	2 (0, 2) 14 (5, 9)	12.5 87.5

Abbreviations: AJCC 7^th^, American Joint Committee on Cancer Staging Manual 7^th^ Edition; GEJ, gastroesophageal junction

The patients were placed in a supine position with thermoplastic immobilization. Intravenous contrast-enhanced CT simulation was performed at 5-mm intervals with a 16-slice Brilliance Big Bore CT (Philips Medical Systems, Cleveland, OH). In all patients, the CT scan was performed from the 6th cervical vertebra to the 5th lumbar vertebra.

As this was an *in silico* planning experiment without actual treatment, data were derived from our ongoing clinical phase II study, which was approved by the ethics committee of our hospital and was registered in clinicaltrials.gov (NCT02312284).

### Definition of clinical target volume and OARs

The delineation of the clinical target volume (CTV) depended on the location of the primary tumor and the guidelines for the involved lymph node regions issued by Japanese Gastric Cancer Association [31], and has been published in our previous study [29]. Generally, the CTV included anastomoses, duodenal stump, and tumor bed (only for stage T4b, if present) and regional lymph nodes. The distal border of the CTV is at the lower border of the left renal vein. The remnant stomach was not routinely included within the radiation field. The PTV consisted of the CTV with a 5-7 mm margin in the radial direction and a 10 mm margin in the superior-inferior direction.

**Figure 4:**
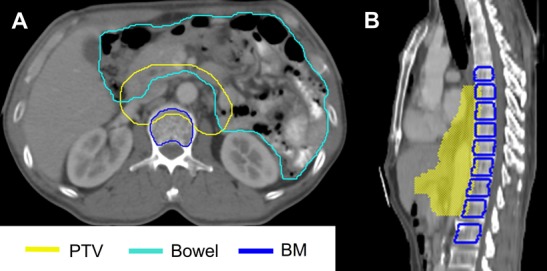
Examples of contours for the bowel and the bone marrow (BM) **a**. The entire bowel cavity including the planning target volume (PTV) and the BM without the layer of compact bone were contoured. **b**. BM volume was defined as the vertebras of where the PTV existed, with an additional one vertebra superior and inferior to the PTV.

We opted to use the content of the entire bowel cavity, including PTV, as a surrogate of the small intestine and the colon (shown in Figure 4a). The bowel was delineated exceeding the lower border of the PTV by 4 slices. BM volume was defined by contouring the vertebras of where the PTV existed, as well as at an additional one vertebra superior and inferior to the PTV (Figure 4b) [20]. Then, we subtracted 1.5 mm from the wall of the BM to exclude the cortex and the entire contents of the medullary canals were contoured (Figure 4a). OARs were contoured as the entire volume including overlapped coverage with the PTV.

Dose constraints for OARs were as follows: V30 < 30% for the liver; V20 < 30% for both kidneys and D_mean_ of < 20 Gy; V30 < 40% for the heart; V10 < 40% and V20 < 20% for both the lungs; and the D1 for the PRV of the spinal cord (adding a uniform margin of 5 mm to the spinal cord) was 40 Gy. The dose for the bowel and BM was set as low dose as possible while keeping the D1 for the bowel less than 50 Gy.

### Treatment planning

To generate comparable plans, one physicist with over 10-year clinical experience designed the IMRT and VMAT plans, while another physicist designed the TOMO plans.

IMRT was developed using the Direct Machine Parameter Optimization algorithm. Five coplanar beams (angles of 20°, 60°, 100°, 180° and 340°), with a step and shoot technique were performed on the Philips Pinnacle planning system, version 9.0 (Philips Radiation Oncology Systems, Fitchburg, WI, USA).

The VMAT plan was optimized in the same planning system as aforementioned. The double arcs with a gantry rotation of 340°−180°−340° were used with a final gantry spacing of 4°. A delivery time of 120 s/arc was used during the optimization.

For the TOMO plan, a field width of 2.5 cm, pitch of 0.287 and modulation factor of 2.2 were used on the TOMO planning system (Hi-Art TomoTherapy 4.1.2, TomoTherapy, Madison, WI, USA).

### Dose comparison

Dose distributions and DVH for the PTV and OARs were compared using the three techniques. The homogeneity index (HI) and conformity index (CI) for the PTV were defined as follows [32]:

$$\begin{array}{l} \text{HI}=\frac{D_{2}}{D_{98}}\\ \text{CI}=\frac{V_{\text{PTV}45}}{V_{\text{PTV}}}\times \frac{V_{\text{PTV}45}}{V_{\text{T}}} \end{array}$$

where Dx represents the minimum dose to the x% of the volume of PTV exposed to the highest dose, V_PTV45_ is the volume of the PTV target covered by 45 Gy, V_PTV_ is the volume of PTV target, and V_T_ is the volume of the body covered by 45 Gy.

The optimal value of HI and CI was 1. The number of MUs required to execute each plan were additionally tabulated.

### Statistical analysis

The data were presented as the mean ± standard deviation with SPSS Version 20.0 (IBM SPSS Inc., Armonk, NY). Based on the Wilcoxon signed rank test, a *p* value < 0.05 was considered to be statistically significant.
